# Antibiotics and antibiotic-associated diarrhea: a real-world disproportionality study of the FDA adverse event reporting system from 2004 to 2022

**DOI:** 10.1186/s40360-023-00710-w

**Published:** 2023-12-04

**Authors:** Haining Huang, Lanfang Li, Mingli Wu, Zhen Liu, Yanyan Zhao, Jing Peng, Xiaolei Ren, Shuai Chen

**Affiliations:** 1 Department of Clinical Pharmacy, Affiliated Hospital of Jining Medical University, Jining, Shandong China; 2 Data Center, Affiliated Hospital of Jining Medical University, Jining, Shandong China; 3 Department of Pathology, Affiliated Hospital of Jining Medical University, Jining, Shandong China

**Keywords:** Antibiotics, Antibiotic-associated diarrhea, The US food and drug administration adverse event reporting system, Pharmacovigilance, Data mining

## Abstract

**Background:**

Our study aimed to assess the risk signals of antibiotic-associated diarrhea (AAD) caused by various antibiotics using real-world data and provide references for safe clinical applications.

**Methods:**

We analyzed data extracted from the FDA Adverse Event Reporting System (FAERS) database, covering the period from the first quarter of 2004 to the third quarter of 2022. We computed the reporting odds ratio (ROR) for each antibiotic or antibiotic class to compare the signal difference. Furthermore, we also examined the differences in the onset times and outcomes of AAD caused by various antibiotics.

**Results:**

A total of 5,397 reports met the inclusion requirements. Almost all antibiotics, except tobramycin and minocycline (ROR 0.98; 95%CI: 0.64–1.51 and 0.42; 95%CI: 0.16–1.11, respectively), showed a significant correlation with AAD. The analysis of the correlation between different classes of antibiotics and AAD revealed that lincomycins (ROR 29.19; 95%CI: 27.06–31.50), third-generation cephalosporins (ROR 15.96; 95%CI: 14.58–17.47), and first/second generation cephalosporins (ROR 15.29; 95%CI: 13.74–17.01) ranked the top three. The ROR values for antibiotics from the same class of antibiotics also varied greatly, with the ROR values for third-generation cephalosporins ranging from 9.97 to 58.59. There were also differences in ROR values between β-lactamase inhibitors and their corresponding β-lactamase drugs, such as amoxicillin-clavulanate (ROR = 13.31; 95%CI: 12.09–14.65) and amoxicillin (ROR = 6.50; 95%CI: 5.69–7.44). 91.35% of antibiotics have an onset time of less than four weeks.

**Conclusions:**

There is a significant correlation between almost all antibiotics and AAD, particularly lincomycins and β-lactam antibiotics, as well as a different correlation within the same class. These findings offer valuable evidence for selecting antibiotics appropriately.

**Supplementary Information:**

The online version contains supplementary material available at 10.1186/s40360-023-00710-w.

## Background

Antibiotic-associated diarrhea (AAD) is defined as diarrhea brought on by taking antibiotics, either while taking them or for up to 8 weeks after antibiotics discontinuation [1]. The excessive use of broad-spectrum antibiotics disrupts the balance of healthy gut bacteria and leads to AAD. Approximately 5% ~ 35% of patients suffer from AAD after receiving antibiotics [2]. The incidence rate of AAD shows an upward trend due to the widespread use of antibiotics. AAD is frequently caused by various pathogenic bacteria, with Clostridium difficile (CD) being responsible for almost one-third of AAD cases [3, 4]. Moreover, a recent meta-analysis comprising 5,496 patients revealed that CD is responsible for 20% of AAD cases among hospitalized patients [5].

CD is a gram-positive, spore-forming, toxin-producing bacillus that is part of the commensal intestinal flora and is widespread in the natural environment [6]. Overuse of some antibiotics can speed up the growth rate of CD, which can influence other bacteria in the gastrointestinal system, leading to inflammation. C. difficile can multiply from either endogenous or exogenous sources once there is an imbalance in the types of organisms present in a person's natural gut flora, known as gut dysbiosis.Clostridium difficile infection (CDI) is a major cause of nosocomial infections, particularly in developed countries [7]. In Europe, where the number of cases is estimated to be 124,000 per year, C. difficile ranked as the sixth most common microorganism causing healthcare-associated infections in the European Prevalence Study of 2016–2017 [8]. The burden of C. difficile also extends to the community, with an estimated 51.9 community-associated infections per 100,000 people attributed to CDI [9]. Pediatric patients who develop postoperative CDI in urologic surgery experience longer hospital stays, higher readmission rates, and increased rates of non-CDI complications [10]. The symptoms of CDI range from mild to profuse diarrhea, severe colitis, and in rare cases, toxic megacolon [4, 11]. Antibiotic exposure, older age, and hospitalization are significant patient-related risk factors for CDI [12, 13]. While almost all antibiotics can lead to CDI, broad-spectrum penicillins and cephalosporins, clindamycin, and fluoroquinolones have a higher risk of inducing CDI [14–16].

The excessive and inappropriate use of antibiotics and the emergence of novel antibiotics in recent years has resulted in an increase in the global incidence rate and severity of AAD. Therefore, further research is needed to assess the relationship between antibiotics and AAD. The current studies are mainly based on retrospective research, while only a few studies using data-mining techniques have specifically examined the reporting correlation between partial antibiotics and AAD [17]. The FDA Adverse Event Reporting System (FAERS) is a database for voluntarily and spontaneously reporting adverse drug effects occurring after marketing [18]. In this study we aimed to extract all reports of AAD following the use of antibiotics from 2004 to September 2022 from the FAERS database, especially for novel antibiotics that have emerged in recent years, and analyzed signals of AAD from all antibiotics or antibiotic classes based on disproportionality analysis. Notably, our study also analyzed the onset times and outcomes of AAD induced by various antibiotics, which have not been previously reported.

## Methods

### Data source

Data was gathered through retrospective pharmacovigilance research using the FAERS database for the first quarter of 2004 to the third quarter of 2021. FAERS is a database that contains adverse event reports, information on medication errors, and product quality concerns that result in adverse events and is intended to enhance the FDA's post-marketing oversight of chemical pharmaceuticals and biological goods. FAERS data contains demographic characteristics, drug information (drug name, active ingredient, drug dose, drug frequency, duration of use), patient outcomes, reporter information, and reaction information.

### Data mining

The search strategy was that within the FAERS database we specified a “Search by Reaction T erm” and looked up the preferred terms (PTs) and then downloaded the raw data. Python (version 3.8) and Postgresql (version 14) were used to clean and normalize FAERS data [19]. All drug-related terms were standardized as drug ingredient names according to RxNorm, and adverse events (AEs) were standardized according to the preferred terms (PTs) and system organ classes (SOCs) in the Medical Dictionary for Regulatory Activities (MedDRA) V 24.0. We looked into the relationships between different antibiotics and AAD using the reporting odds ratio (ROR), which is based on the principles of disproportionality analysis. A two-by-two contingency table (Table 1) of reported event counts for a specific drug and other drugs was created to calculate ROR. The calculation and criteria of the algorithm are as follows: Table 1 [18, 20, 21]. AAD cases were recognized by searching using the Medical Dictionary for Regulatory Activities (MedDRA) (version 24.1), and PTs were displayed in Table 2. The drug role code was identified as the primary suspect drug (PS) in the entire dataset.

**Table 1:** Two-by-two contingency table for disproportional analysis

	AAD	All other adverse drug reactions	Total
antibiotics	a	b	a + b
Other drugs	c	d	c + d
Total	a + c	b + d	a + b + c + d

ROR = ad/bc, 95%CI = e^ln(ROR)^.^±1.96^ $\sqrt{\frac{1}{\mathrm{a}}}+\frac{1}{\mathrm{b}}+\frac{1}{\mathrm{c}}+\frac{1}{\mathrm{d}}$ (criteria: the lower 95%Cl > 1,a ≥ 3)

*ROR* Reporting odds ratio, *AAD* Antibiotic-associated diarrhea.

**Table 2:** MedDRA PTs used to search AAD events in FAERS database

PTs-code	PTs-name
10,009,657	Clostridium difficile colitis
10,037,128	Pseudomembranous colitis
10,052,815	Antibiotic associated colitis
10,058,305	Clostridium colitis
10,058,852	Clostridium bacteraemia
10,061,043	Clostridial infection
10,070,027	Clostridium test positive
10,078,496	Clostridial sepsis

PTs, the preferred terms

In addition, we extracted the onset times for all involved antibiotics and calculated the onset times in groups. We also extracted the onset times of most common antibiotics from these of all involved antibiotics and the onset times were shown individually as median (Q1, Q3). The period between the beginning of antibiotic use and the occurrence of AE is used to calculate the onset times [22]. Severe outcomes events induced by antibiotics included “Death” (grade 5), “Life-Threatening” (grade 4), “Disability” (grade 4) and Congenital Anomaly “(grade 4) [23, 24]. “Hospitalization-Intial or Prolonged” (grade 3) was considered to be moderate outcomes events, and the rest was classified as mild outcomes events. The proportion of reports with severe outcomes was computed by dividing the number of severe outcomes events by the total number of outcomes events. The mortality rate of AAD was calculated by dividing the number of death outcomes events by the total number of reports.

### Statistical analysis

The clinical characteristics of patients with AAD derived from the FAERS database were summarized using categorical variables presented as frequency and percentage. A quartile assay was used to display the onset times of AAD. A disproportionality analysis was carried out by counting ROR and a corresponding 95% confidence interval (CI) for the relationship between AAD and drugs [25]. The lower limit of the 95% CI above 1.0 was considered to be statistically significant [25]. A higher ROR suggested a stronger reporting association between AAD and drugs.

## Results

### Descriptive analysis

**Fig. 1:**
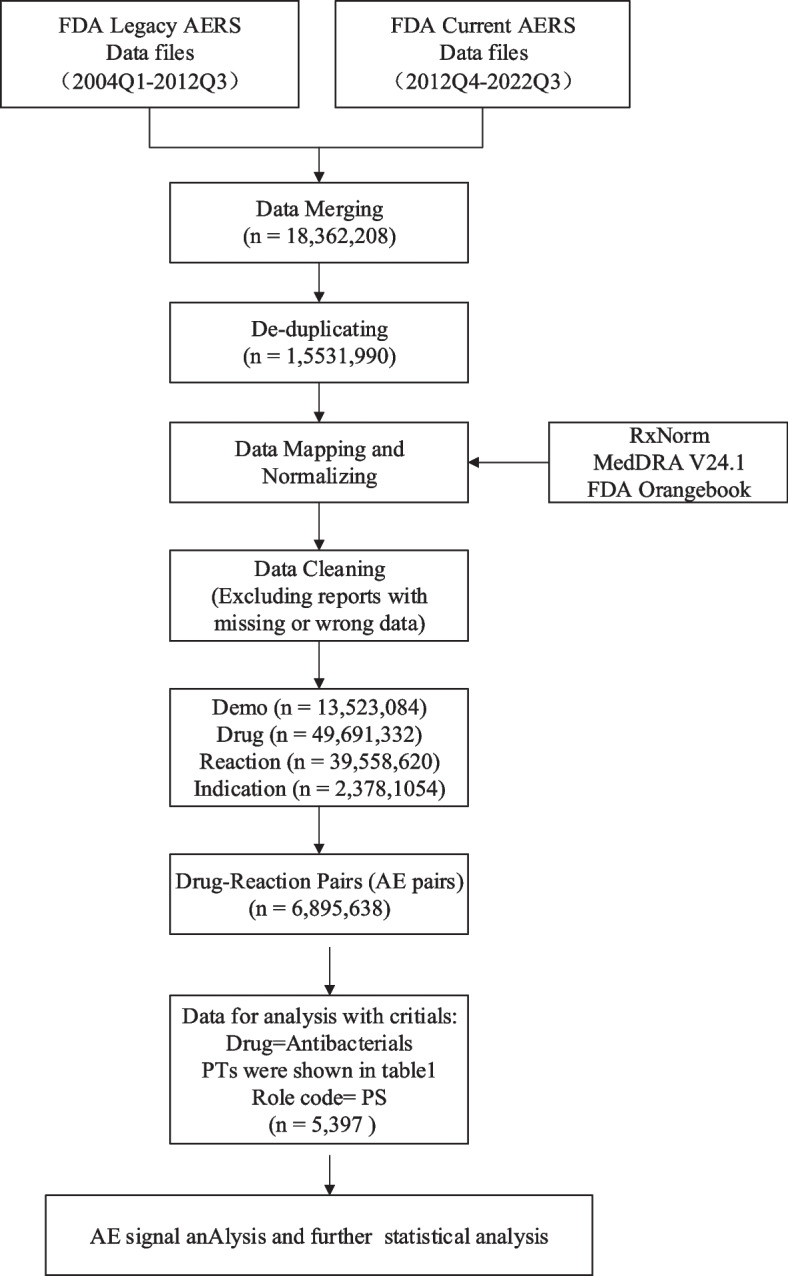
Flow diagram of data extraction and cleaning

 FAERS database from 2004 to September 2022 contains 18,362,208 AE reports. After data cleaning and matching, 6,895,638 AE reports were included in future research. 5397 reports of AAD following the use of antibiotics were acquired (Fig. 1). The clinical characteristics of these patients were summarized in Table 3. Patients over 65 years old have a larger proportion than other age groups (43.21% vs. 34.59%, 17.92%, 4.28%). Females accounted for a larger proportion than males in all reports (51.49% vs. 36.85%). Cases were mainly reported by the physician (31.52%) and health-professional (22.48%), and were mostly from North America (48.36%), and Europe (37.54%). The number of reported cases remained stable at around 200 cases from 2004 to 2012, while more cases were reported from 2013 and the number of reported cases in 2019 is the highest.

**Table 3:** Clinical characteristics of target patients with AAD

Characteristics	Reports, n (%)
Patient age(year)	
< 18	231 (4.28%)
18–64	1867 (34.59%)
≥ 65	2332 (43.21%)
Unkown	967 (17.92%)
Patient gender	
Male	1989 (36.85%)
Female	2779 (51.49%)
Unkown	629 (11.65%)
Reporter	
Consumer	1054 (19.53%)
Lawyer	14 (0.26%)
Other health-professional	1213 (22.48%)
Pharmacist	885 (16.40%)
Physician	1701 (31.52%)
Unkown	530 (9.82%)
Continent	
Africa	25 (0.46%)
Asian	329 (6.10%)
Europe	2026 (37.54%)
North America	2610 (48.36%)
Oceania	18 (0.33%)
South America	49 (0.91)
Unkown	340 (6.30%)
Year	
2004	207 (3.84%)
2005	210 (3.89%)
2006	231 (4.28%)
2007	206 (3.82%)
2008	266 (4.93%)
2009	294 (5.45%)
2010	224 (4.15%)
2011	205 (3.80%)
2012	172 (3.19%)
2013	352 (6.52%)
2014	294 (5.45%)
2015	324 (6.00%)
2016	306 (5.67%)
2017	313 (5.80%)
2018	352 (6.52%)
2019	440 (8.15%)
2020	322 (5.97%)
2021	414 (7.67%)
2022	265 (4.91%)

*AAD* Antibiotic-associated diarrhea, *n* Number of reports

### Disproportionality analysis

**Fig. 2:**
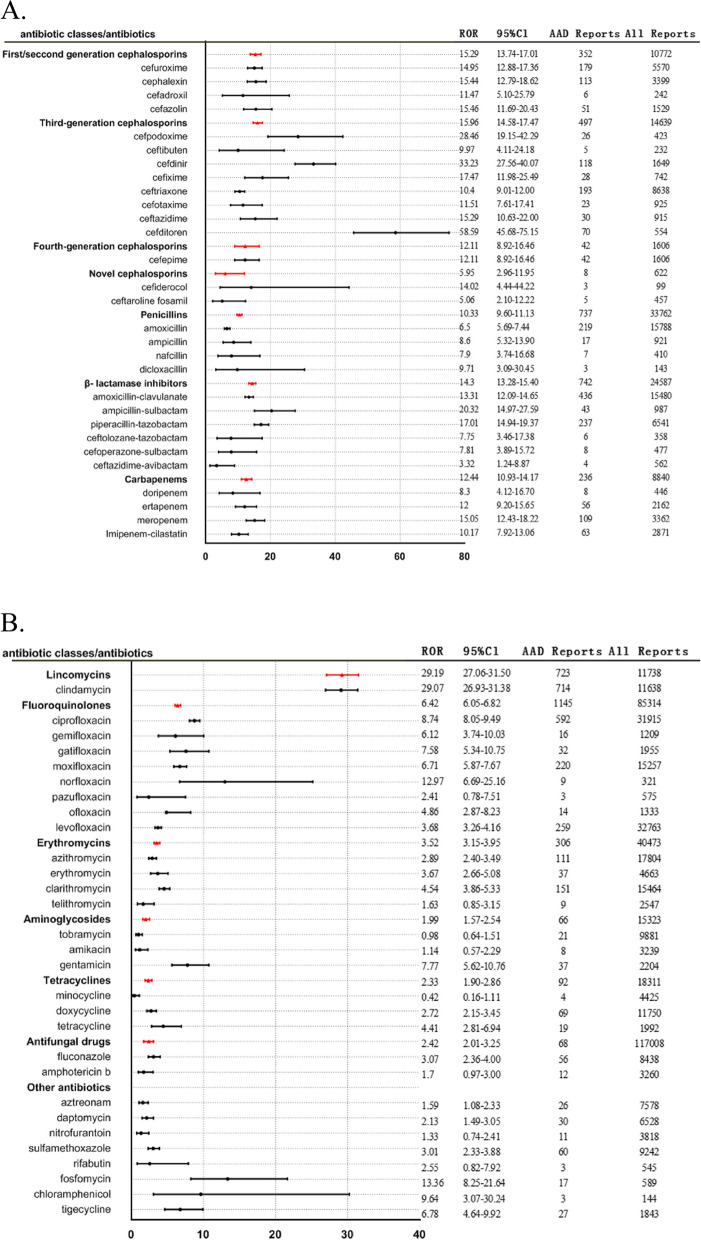
ROR value for AAD with antibiotics or antibiotics classes. A. β-lactam antibiotics; B. other antibiotics. ROR, reporting odds ratio; AAD, antibiotic-associated diarrhea; Cl, confidence interval

We analyzed signals of AAD from all antibiotics based on the criteria for ROR and recorded the results in Fig. 2 (A, B). Our findings revealed that almost all antibiotics have a correlation with AAD except for tobramycin (ROR 0.98; 95%CI: 0.64–1.51) and minocycline (ROR 0.42; 95%CI: 0.16–1.11). Cefditoren (ROR = 58.59; 95%CI: 45.68–75.15), cephradine (ROR = 42.06; 95%CI: 12.90–137.14), lincomycin (ROR = 41.65; 95%CI: 21.05–82.39) had a higher ROR. The ROR values of β- lactamase inhibitors were different from corresponding β- lactamase drugs, such as amoxicillin-clavulanate (ROR = 13.31; 95%CI: 12.09–14.65) and amoxicillin (ROR = 6.50; 95%CI: 5.69–7.44), ampicillin-sulbactam (ROR = 20.32; 95%CI: 14.97–27.59) and ampicillin (ROR = 8.60; 95%CI: 5.32–13.90), ceftazidime-avibactam (ROR = 3.32; 95%CI: 1.24–8.87) and ceftazidime (ROR = 15.29; 95%CI: 10.63–22.00).

The results of cephradine (ROR = 42.06, 95%CI: 12.90–137.14) and floxacillin (ROR = 34.92, 95%CI: 12.64–96.48) were too large to display perfectly in the forest plots. As demonstrated in Fig. 2, antibiotics were categorized into the following groups: first/second-generation cephalosporins, third-generation cephalosporins, fourth-generation cephalosporins, novel cephalosporins, penicillins, β- lactamase inhibitors, carbapenems, lincomycins, fluoroquinolones, erythromycins, aminoglycosides, tetracyclines, other antibiotics, antifungal drugs, and the ROR values were calculated by category. The analysis of the correlation between different antibiotics classes and AAD showed the following ranking: lincomycins (ROR = 29.19; 95%CI: 27.06–31.50) > third-generation cephalosporins (ROR = 15.96; 95%CI: 14.58–17.47) > first/second generation cephalosporins (ROR = 15.29; 95%CI: 13.74–17.01) > β- lactamase inhibitors (ROR = 14.30; 95%CI: 13.28–15.40) > carbapenems (ROR = 12.44; 95%CI: 10.93–14.17) > fourth-generation cephalosporins (ROR = 12.11; 95%CI: 8.92–16.46) > penicillins (ROR = 10.33; 95%CI: 9.60–11.13) > fluoroquinolones (ROR = 6.42; 95%CI: 6.05–6.82) > novel cephalosporins (ROR = 5.95; 95%CI: 2.96–11.95) > erythromycins (ROR = 3.52; 95%CI: 3.15–3.95) > tetracyclines (ROR = 2.33; 95%CI: 1.90–2.86) > aminoglycosides (ROR = 1.99; 95%CI: 1.57–2.54).

### Time to onset of AAD

The onset times of AAD for all involved antibiotics are summarized in Table 4 and the onset times of AAD induced by part antibiotic was shown as median (Q1, Q3) separately in additional files 1: Table S1. The number of AAD reports with an onset time less than one week was 1336 (52.47%) and the number with an onset time from one week to four weeks was 990 (38.88%), of which 91.35% were reported with an onset time less than 4 weeks. The median time to onset was within 14 days for most antibiotics except for doxycycline (15 days). The shortest median time to onset was 3 (2–10) days for cefoperazone-sulbactam and 3 (1–6) days for cefazolin, respectively.

**Table 4:** Onset times of AAD associated with all involved antibiotics

Onset time	AAD Reports (%)
< 1 week	1336(52.47%)
1-4 week	990(38.88%)
4-8 week	131(5.15%)
8-12 week	38(1.49%)
> 12 week	51(2.00%)

*AAD*, Antibiotic-associated diarrhea

### Outcomes due to AAD

**Fig. 3:**
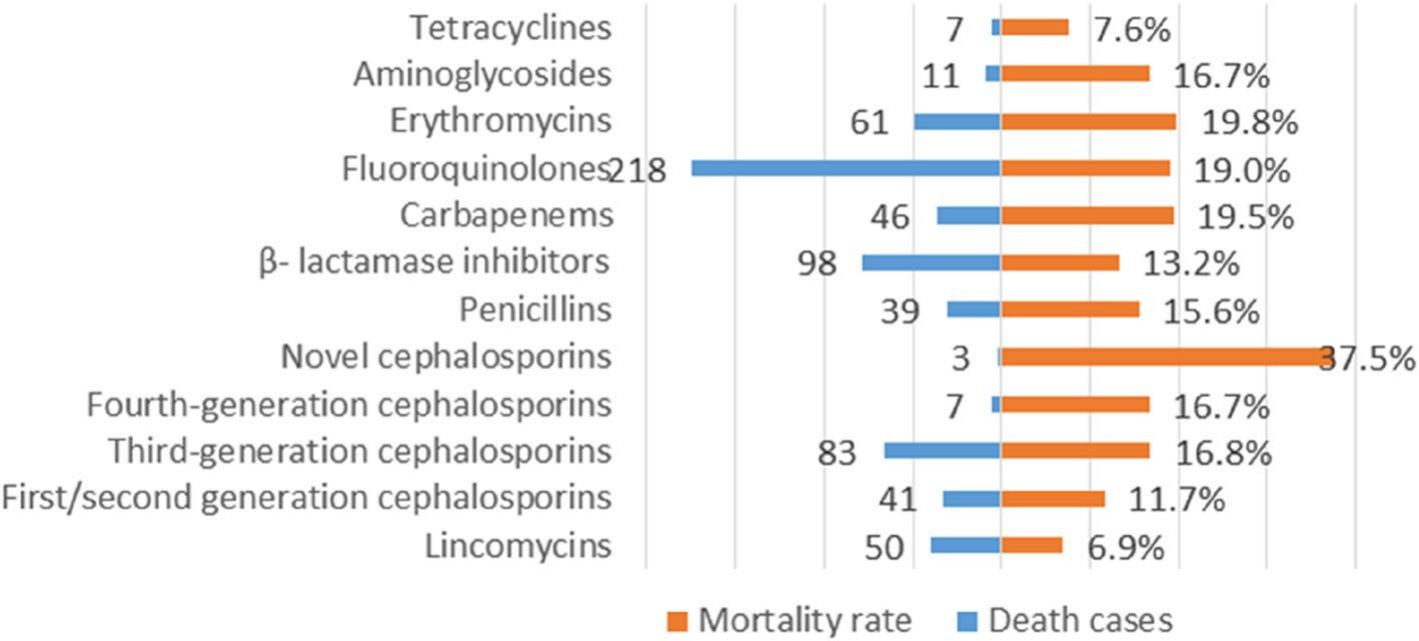
Mortality rate for AAD associated with antibiotics

To evaluate the prognosis of patients with AAD induced by antibiotics, we calculated the proportion of each outcome event and the mortality rate, and the results were shown in Table 5, Fig. 3, and additional files 1: Table S2. Our results indicated that serious cases account for 23.8% of all AAD cases, while mild and moderate cases account for the majority of all AAD cases. In the antibiotic class, novel cephalosporins had the highest mortality rate (37.5%), and the lowest for lincomycins (6.9%). In all antibiotics, chloramphenicol (66.7%), cefiderocol (66.7%), lincomycin (55.6%) and ceftazidime-avibactam (50.0%) had the highest mortality rate, and the lowest for cefadroxil, floxacillin pazufloxacin mesilate, tetracycline, minocycline, rifabutin (0.0%).

**Table 5:** Outcomes events of AAD

Outcomes events	Reports (%)
Death (grade 5)	805(12.03%)
Life-Threatening (grade 4)	532(7.95%)
Disability (grade 4)	253(3.78%)
Congenital Anomaly (grade 4)	3(0.04%)
Hospitalization-Intial or Prolonged(grade3)	4381(65.47%)
Required Intervention to Prevent Permanent (grade 2)	138(2.06%)
Other Serious (Important Medical Event) (grade 1)	580(8.67%)

*AAD* Antibiotic-associated diarrhea

## Discussion

In recent years, AAD has become a global concern due to the emergence of highly virulent strains and the widespread use of antibiotics such as clindamycin and cephalosporins [26]. Studies have shown that the incidence rate of AAD is increasing every year in China and other countries. Research on AAD and CDI has gained attention recently, with findings supporting the strong relationship between AAD and CD [27]. Clostridium difficile is a major cause of infectious diarrhea during antibiotic administration. Given the constant development and widespread use of novel antibiotics, understanding the connection between antibiotics and AAD is crucial.

However, the previous real-world study conducted from 2015 to 2017 only included a small number of antibiotics [17]. As a result, the data needs to be updated. Our study screened more reports of AAD following the use of antibiotics from 2004 to September 2022, including novel antibiotics in recent years. Above all, the onset times and outcomes of AAD induced by various antibiotics have not been previously reported.

Our study represents the largest data collection of real-world research to date, using data collected in the FAERS database to examine differences in AAD produced by various antibiotics in terms of correlation, onset time, prognosis, and more. Our findings showed that patients aged 65 years or older had a higher ROR value, indicating that their probability of developing AAD was higher. We hypothesized that the reason why elderly individuals are more susceptible to AAD may be due to a weakened immune response, resulting in a poorer ability to produce a serum IgG antitoxin A antibody response to C [28]. Additionally, females accounted for a larger proportion than males in all reports (51.49% vs. 36.85%). This result suggests that females may be more susceptible to AAD than males, potentially due to differences in gut flora between genders [29]. These findings are consistent with recent research in the relevant literature [17].

The study found that nearly all antibiotics were strongly associated with AAD events, consistent with a clinical retrospective study [30]. When the ROR value was calculated for each antibiotic, cefditoren (ROR = 58.59; 95%CI: 45.68–75.15), cephradine (ROR = 42.06; 95%CI: 12.90–137.14), and lincomycin (ROR = 41.65; 95%CI: 21.05–82.39) had the highest ROR values. When the ROR value was calculated by antibiotic class, lincomycins (ROR = 29.19; 95%CI: 27.06–31.50) had the highest value, with most β-lactam antibiotics having higher ROR values, as described in previous studies [17]. Notably, the study revealed that the rank correlation between the different classes of antibiotics and AAD was as follows: lincomycins > third-generation cephalosporins > first/second-generation cephalosporins > β-lactamase inhibitors > carbapenems > fourth-generation cephalosporins > penicillins > fluoroquinolones > novel cephalosporins > erythromycins > tetracyclines > aminoglycosides. In a meta-analysis, the ranking was as follows: third-generation cephalosporins > clindamycin > second-generation cephalosporins > fourth-generation cephalosporins > carbapenems > trimethoprim-sulfonamides > fluoroquinolones > penicillin combinations [31]. Third-generation cephalosporins had the highest ROR value compared to β-lactam antibiotics, while new cephalosporins had the lowest. The results were consistent with previous research indicating that broad-spectrum antibiotics such as lincomycin, cephalosporin, and penicillin were more likely to result in AAD [32–34]. This may be due to C. difficile isolates being completely resistant to clindamycin, most cephalosporins, and penicillin. There is no comparison with cephalosporins, although previously published studies suggested that fluoroquinolones were similarly significant risk factors for causing AAD [26, 35, 36]. The probable explanation for this finding is that nearly all fluoroquinolones exhibit a high minimum inhibitory concentration (MIC) against C. difficile, thereby leading to a high resistance rate. In our study, ROR for AAD with fluoroquinolones was 6.42, implying that the signal of AAD induced by fluoroquinolones was significantly lower than that of β-lactam antibiotics, which has not been previously reported. We hypothesize that the reason for this may be that fluoroquinolones are not as widely utilized as beta-lactam antibiotics due to their restricted usage to avoid adverse effects.

The ROR value for AAD caused by the same class of antibiotics also varied greatly, with the ROR values for first/second-generation cephalosporins ranging from 11.47 to 42.06, those for third-generation cephalosporins ranging from 9.97 to 58.59, and those for penicillins ranging from 6.50 to 34.92. Therefore, the degree of AAD induced by the same class of antibiotics can differ. These findings provide a strong foundation for choosing antibiotics.

A recent study found that patients treated with enzyme inhibitor antibiotics had a significantly higher incidence of AAD (35.36% vs. 21.43%) than those treated with non-enzyme inhibitor antibiotics (P = 0.013) [34, 37]. This could be attributed to the frequent use of enzyme inhibitor antibiotics in the treatment of multidrug-resistant bacteria among critically ill patients who require extended treatment periods. Studies have shown a correlation between the duration of enzyme inhibitor antibiotic therapy and the occurrence of AAD in critically ill patients. Additionally, prolonged use of enzyme inhibitor antibiotics may lead to alterations in the intestinal microbiota, thereby increasing the likelihood of AAD. [34]. Our findings similarly revealed differences in ROR values between β-lactamase inhibitors and their corresponding β-lactamase drugs. For example, amoxicillin-clavulanate (ROR = 13.31; 95%CI: 12.09–14.65) and amoxicillin (ROR = 6.50; 95%CI: 5.69–7.44), ampicillin-sulbactam (ROR = 20.32; 95%CI: 14.97–27.59) and ampicillin (ROR = 8.60; 95%CI: 5.32–13.90), ceftazidime-avibactam (ROR = 3.32; 95%CI: 1.24–8.87) and ceftazidime (ROR = 15.29; 95%CI: 10.63–22.00).

The abuse of antibiotics, particularly broad-spectrum antibiotics, is commonly believed to be the main cause of AAD. It is noteworthy that antifungal drugs are also included in broad-spectrum antibiotics. A recent retrospective study has revealed a higher incidence of antifungal-associated diarrhea (AAD) in patients within the intensive care unit who were treated with antifungals. This outcome is likely attributed to the fact that antifungals are commonly administered alongside other antibiotics, increasing the likelihood of inducing AAD [37, 38]. In our study, amphotericin b and fluconazole were the existing antifungals in the antibiotics that met the inclusion criteria. Their ROR values and 95% Cl were (ROR = 1.70, 95%CI: 0.97–3.00) and (ROR = 3.07, 95%CI: 2.36–4.00), respectively, suggesting that these two antifungal drugs were associated with AAD.

The period between drug intake and symptom onset varies but is typically short [39]. A previous study published in 2012 showed that the most contagious times for potential donors to support the transmission of C. difficile were ≤ 1 week (65%), ≤ 4 weeks (82%), and > 8 weeks (only 10%) [40]. Our study found that the onset times of AAD associated with all involved antibiotics were ≤ 1 week (52.47%), ≤ 4 weeks (91.35%), and > 8 weeks (3.49%), which was consistent with previous study results. Notably, we also examined the separate onset times of each antibiotic, and the result was that the onset times of AAD caused by the same class of drugs also varied. The onset times of AAD induced by cephalosporins ranged from 3 days (cefazolin) to 8.5 days (ceftazidime). This may be due to the varying abilities of different antibiotics to disrupt the intestinal flora or inhibit the activity of C. difficile, resulting in differing periods of AAD onset. We should therefore analyze the onset times of AAD caused by antibiotics separately for each drug.

Most cases of antibiotic-associated diarrhea (AAD) are mild and self-limiting, typically resolving within 5 to 10 days after discontinuing antibiotics therapy. However, one type of AAD called Clostridioides difficile infection (CDI) can result in severe gastrointestinal disease, ranging from diarrhea and fever to colitis, toxic megacolon, multi-organ failure, or death [11]. Hence, it is essential to monitor the prognosis of AAD caused by antibiotics. This study utilized the FAERS database to determine the real-world prognosis of AAD for the first time. The findings indicated that mild and moderate AAD cases constituted the majority of cases, consistent with previous reports [11]. Yet, death due to AAD still occurs, with a high mortality rate associated with C. difficile antibiotic diarrhea (CDAD), particularly in patients over 65 years with underlying or severe diseases [41]. The study discovered that 12.3% of AAD cases were classified as "Death" cases (grade 5). In the United States, CDI has an approximate incidence rate of 453,000 cases and 29,000 deaths in 2011 [9], calculating a mortality rate of 6.4%. The population included in our study came from a variety of countries, some of which had lax antibiotics regulation, which could account for the mortality rate of our research findings. Another finding is that lincomycins had the lowest mortality rate (6.4%) while new cephalosporins had the highest mortality rate (37.5%). This result may be related to the patient's own disease and the management of antibiotics. Novel antibiotics are frequently subject to strict regulations and limited to patients experiencing severe infections. This patient population typically presents with multiple comorbidities, complex diseases, and a high mortality rate.

### Limitations

Although the study had advantages in data mining utilizing the FAERS database, it had inherent limitations, such asthe inability to establish whether the drug caused the event [42]. Additionally, some antibiotics could be combined with other drugs, increasing the probability of AAD. Despite these limitations, this study suggests that FAERS serves as a pharmacovigilance tool for alerting individuals to the varying degrees of AAD resulting from different antibiotics.

## Conclusion

This study represents the first pharmacovigilance investigation to examine all antibiotics in the FAERS database, identifying potential links between antibiotics and AAD, and comparing the timeframes and outcomes of AAD triggered by different antibiotics.

Our comprehensive and systematic retrospective analysis of the FAERS database revealed a significant correlation between most post-marketing antibiotics and AAD and a different correlation within the same class, which has wider implications for antibiotic stewardship. When compared to other antibiotic classes, lincomycins and β-lactam antibiotics were more strongly associated with AAD, and within the β-lactam antibiotic category, third-generation cephalosporins had a higher risk of causing AAD. Our findings also indicated that female patients and those over 65 years of age had a higher risk of developing AAD. We discussed the time-to-onset and outcomes of AAD induced by antibiotics, providing valuable insights for clinical practice and adverse drug reaction monitoring. Overall, our study offers valuable evidence to inform clinical interventions for the management of AAD caused by antibiotics.

### Supplementary Information

**Additional file 1: Table S1. **Onset times of AAD associated with each antibiotic. **Table S2.** Mortality rate for AAD associated with antibiotics.

## Data Availability

FDA Adverse Event Reporting System data are available at https://www.fda.gov/drugs/questions-and-answers-fdas-adverse-event-reporting-system-faers/fda-adverse-event-reporting-system-faers-public-dashboard. The 'Search' tab was then selected, and the remaining information can be found in the Methods section of the manuscript.
