# Supplementary material for: Antibiotics and antibiotic-associated diarrhea: a real-world disproportionality study of the FDA adverse event reporting system from 2004 to 2022
Source: BMC Pharmacol Toxicol. 2023 Dec 4;24:73. doi: 10.1186/s40360-023-00710-w (PMC10694877; doi:10.1186/s40360-023-00710-w)
Supplement: Supplementary file 1 — Additional file 1: Table S1. Onset times of AAD associated with each antibiotic. Table S2. Mortality rate for AAD associated with antibiotics. [file 40360_2023_710_MOESM1_ESM.docx]

**Table S1** Onset times of AAD associated with each antibiotic.

| **antibiotics** | **Reports** | **M（Q1-Q3）（d）** |
| --- | --- | --- |
| amoxicillin | 122 | 8 (4-34) |
| Amoxicillin-clavulanate | 299 | 8 (3-11) |
| piperacillin-tazobactam | 138 | 7 (3-13) |
| cephalexin | 59 | 8 (4-16) |
| cefepime | 16 | 6 (3.3-18.3) |
| cefpodoxime | 19 | 5 (4-11) |
| cefdinir | 64 | 7.5 (3-10) |
| cefuroxime | 94 | 7 (3.8-8) |
| cefixime | 18 | 6 (4-8) |
| cefoperazone-sulbactam | 7 | 3 (2-10) |
| ceftriaxone | 89 | 6 (3-10.5) |
| cefotaxime | 5 | 6 (4.5-24) |
| ceftazidime | 18 | 8.5 (4.5-10) |
| cefazolin | 13 | 3 (1-6) |
| meropenem | 45 | 11 (6-23.5) |
| imipenem-cilastatin | 49 | 8 (3-16) |
| erythromycin | 8 | 5.5 (4-11.5) |
| clarithromycin | 79 | 6 (3-8) |
| azithromycin | 38 | 4 (2-5.3) |
| ciprofloxacin | 236 | 8 (4-15) |
| moxifloxacin | 118 | 6.5 (2-10) |
| levofloxacin | 102 | 8 (4-12.3) |
| doxycycline | 36 | 15 (7-53) |
| metronidazole | 122 | 8 (3-13.3) |
| clindamycin | 393 | 8 (4-11) |

AAD, antibiotic-associated diarrhea; M, Median; Q1, First quartile; Q3, Third quartile; d, days.

**Table S2** Mortality rate for AAD associated with antibiotics.

| **Antibiotics Class/Antibiotics** | **AAD reports (n)** | **Death cases (n)** | **Mortality rate (%)** |
| --- | --- | --- | --- |
| **Lincomycins** | 723 | 50 | 6.92 |
| lincomycin | 9 | 5 | 55.56 |
| clindamycin | 714 | 45 | 6.3 |
| **First/second-generation cephalosporins** | 352 | 41 | 11.65 |
| cefazolin | 51 | 2 | 3.92 |
| cefadroxil | 6 | 0 | 0 |
| cephradine | 3 | 1 | 33.33 |
| cephalexin | 113 | 12 | 10.62 |
| cefuroxime | 179 | 26 | 14.53 |
| **Third-generation cephalosporins** | 493 | 83 | 16.84 |
| cefditoren | 70 | 3 | 4.29 |
| ceftazidime | 30 | 6 | 20 |
| cefotaxime | 23 | 8 | 34.78 |
| ceftriaxone | 193 | 56 | 29.02 |
| cefixime | 28 | 1 | 3.57 |
| cefdinir | 118 | 6 | 5.08 |
| ceftibuten | 5 | 1 | 20 |
| cefpodoxime | 26 | 2 | 7.69 |
| **Fourth-generation cephalosporins** | 42 | 7 | 16.67 |
| cefepime | 42 | 7 | 16.67 |
| **Novel cephalosporins** | 8 | 3 | 37.5 |
| ceftaroline fosamil | 5 | 1 | 20 |
| cefiderocol | 3 | 2 | 66.67 |
| **Penicillins** | 250 | 39 | 15.6 |
| dicloxacillin | 3 | 1 | 33.33 |
| nafcillin | 7 | 3 | 42.86 |
| floxacillin | 4 | 0 | 0 |
| ampicillin | 17 | 4 | 23.53 |
| amoxicillin | 219 | 31 | 14.16 |
| **β- lactamase inhibitors** | 743 | 98 | 13.19 |
| ceftazidime-avibactam | 4 | 2 | 50 |
| cefoperazone-sulbactam | 8 | 3 | 37.5 |
| ceftolozane-tazobactam | 6 | 1 | 16.67 |
| piperacillin-tazobactam | 237 | 32 | 13.5 |
| ampicillin-sulbactam | 45 | 8 | 17.78 |
| amoxicillin-clavulanate | 443 | 52 | 11.74 |
| **Carbapenems** | 236 | 46 | 19.49 |
| Imipenem-cilastatin |  | 14 | 22.22 |
| meropenem | 109 | 28 | 25.69 |
| ertapenem | 56 | 3 | 5.36 |
| doripenem | 8 | 1 | 12.5 |
| **Fluoroquinolones** | 1145 | 218 | 19.04 |
| levofloxacin | 259 | 55 | 21.24 |
| ofloxacin | 14 | 3 | 21.43 |
| pazufloxacin mesilate | 3 | 0 | 0 |
| norfloxacin | 9 | 2 | 22.22 |
| moxifloxacin | 220 | 37 | 16.82 |
| gatifloxacin | 32 | 2 | 6.25 |
| gemifloxacin | 16 | 2 | 12.5 |
| ciprofloxacin | 592 | 117 | 19.76 |
| **Erythromycins** | 308 | 61 | 19.81 |
| telithromycin | 9 | 1 | 11.11 |
| clarithromycin | 151 | 34 | 22.52 |
| erythromycin | 37 | 5 | 13.51 |
| azithromycin | 111 | 21 | 18.92 |
| **Aminoglycosides** | 66 | 11 | 16.67 |
| gentamicin | 37 | 7 | 18.92 |
| amikacin | 8 | 2 | 25 |
| tobramycin | 21 | 2 | 9.52 |
| **Tetracyclines** | 92 | 7 | 7.61 |
| tetracycline | 19 | 0 | 0 |
| doxycycline | 69 | 7 | 10.14 |
| minocycline | 4 | 0 | 0 |
| **Other antibiotics** |  |  |  |
| tigecycline | 27 | 2 | 7.41 |
| chloramphenicol | 3 | 2 | 66.67 |
| fosfomycin | 17 | 1 | 5.88 |
| rifabutin | 3 | 0 | 0 |
| sulfamethoxazole | 60 | 3 | 5 |
| nitrofurantoin | 11 | 1 | 9.09 |
| daptomycin | 30 | 8 | 26.67 |
| aztreonam | 26 | 2 | 7.69 |
| **Antifungal drugs** | 68 | 10 | 14.71 |
| amphotericin b | 12 | 3 | 25 |
| fluconazole | 56 | 7 | 12.5 |

AAD, antibiotic-associated diarrhea; n, number of reports.
